# Address of Welcome

**Published:** 1912-07

**Authors:** Thomas H. Jeffries

**Affiliations:** Atlanta, Ga.


### Journal-Record of Medicine

**Successor to Atlanta Medical and Surgical Journal, Established 1855 and Southern Medical Record, Established 1870.**

**OWNED BY THE ATLANTA MEDICAL JOURNAL CO.**

**Published Monthly**

*Official Organ Fulton County Medical Society, State Examining Board, Presbyterian Hospital, Atlanta, Birmingham and Atlantic Railroad Surgeons' Association, Chattahoochee Valley Medical and Surgical Association, Etc.*

EDGAR G. BALLENGER, M. D., Editor.
BERNARD WOLFF, M. D., Supervising Editor.
A. W. STIRLING, M. D., C. M., D. P. H., J. S. HURT, B. Ph., M. D
GEO. M. NILES, M. D., W. J. LOVE, M. D., (Ala.); Associate Editors.
E. W. ALLEN, Business Manager.

COLLABORATORS

Dr. W. F. WESTMORLAND, General Surgery.
F. W. McRAE, M. D., Abdominal Surgery.
H. F. HARRIS, M. D., Pathology and Bacteriology.
E. B. BLOCK, M. D., Diseases of the Nervous System.
MICHAEL HOKE, M. D., Orthopedic Surgery.
CYRUS W. STRICKLER, M. D., Legal Medicine and Medical Legislation.
E. C. DAVIS, A. B., M. D., Obstetrics.
E. G. JONES, A. B., M. D., Gynecology.
R. T. DORSEY, Jr., B. S. M. D., Medicine.
L. M. GAINES, A. B., M. D., Internal Medicine.
GEO. C. MIZELL, M. D., Diseases of the Stomach and Intestines.
L. B. CLARKE, M. D., Pediatrics.
EDGAR PAULIN, M. D., Opsonic Medicine.
THEODORE TOEPEL, M. D., Mechano Therapy.
R. R. DALY, M. D., Medical Society.
A. W. STIRLING, M. D., etc., Diseases of the Eye, Ear, Nose and Throat.
BERNARD WOLFF, M. D., Diseases of the Skin.
E. G. BALLENGER, M. D., Diseases of the Genito-Urinary Organs.

VOL. LIX. JULY 1912 No. 4

### Edition of the Chattahoochee Valley

### MEDICAL AND SURGICAL ASSOCIATION

### ADDRESS OF WELCOME.

BY HON. THOS. H. JEFFRIES, ATLANTA, GA.

Mr. President and Gentlemen of the Chattahoochee Valley Medical and Surgical Association:

It is with a feeling of gratitude that I appear before you today to extend to you the courtesies and hospitality of this matchless city of the southland.

It has been my pleasure to be a citizen of this city for many years; here I first saw the light of life and upon my advent the first person I met was an honored member of your profession. I have a grateful interest in this Convention, too, because some of the honorable gentlemen who sit before me with patience and fidelity taught, and with generosity, gave my last born son the right to practice medicine.

The origin of the Medical profession is shrouded in mystery. The mythological god whose worship gave rise to the science of medicine was only the deification of a human ideal which future generations developed into the most beneficent science known to man.

I was amused, but not surprised, to find that the modern day physicians have in one particular at least, been loyal to first principles, and follows the practice of the priests of Aesculapius in that, when the patient is cured they "take a statement of the case and require an offering to the god," particularly the offering.

The medical is perhaps the most technical of all the profession, requiring a keener discrimination of the exact meaning of words and phrases to attain definite knowledge. This is forcefully illustrated by the Irishman who had called a physician. The doctor told him that his ailment was *"of long standing,"* whereupon, Pat replied, "Begorra, it must be convalescin' thin, for the last doctor I called said it was *deep seated."*

This, my friends, is illustrative of the cordiality and sincerity of Atlanta's welcome to you to-day. Atlanta has been welcoming people for a long time, the habit is "of long standing" and is deep seated, has indeed become chronic without hope of convalescence.

For more than half a century Atlanta has stood with outstretched hands and smiling faces, bidding welcome to good men and true men of every calling. Welcoming those who were her guests for the hour or the day, welcoming those who came to stay, welcoming everything that promised good to the city or strength to its magic growth. Somtimes, perhaps, in the ardor of her hospitality, she has not been as discriminating as she should have been, but in the hope that she might "entertain angels unawares" she has been exceedingly generous.

This spirit of generosity and kindness is strikingly illustrated by a little incident in the life of one of our citizens attached to the Fire Department. It was his custom each day, in the early morning, to leave the engine house and go to his breakfast at his home, some blocks away.

One morning as he emerged from his cosy quarters he found the sidewalks and streets covered with a great coat of sleet. The Ice King had been on a rampage the night before. The streets were not only covered with sleet, but the ice, clinging to the overhanging wires and boughs of the trees, had crushed many of them to the earth, until our fireman found great difficulty in making his way homeward. As he cautiously picked his way among the fallen wires and boughs, he was startled by a peculiar noise amid the fallen boughs nearby. Looking, he beheld a monster rattlesnake pinioned beneath a fallen bough. His first impulse was to seize a stick and crush out its life, but the serpent looked at him so innocently that his generous spirit prevailed, and instead of killing the snake he lifted the bough from its prostrate form and resumed his journey. Imagine his surprise when, looking back, the serpent followed him. Again he raised a stick to bat out his life. The serpent coiled up and lifting high his head with a look filled with earnest appeal begged him to stay his hand. Again he threw down his weapon and hastened home. The snake followed and slipped in by him as he opened the door, and coiled himself in a cosy corner under the bed. When the snake had thoroughly warmed he began to move about the premises in search of food. He became such a terror to the rats and mice about the place that the fireman concluded that he had added a very valuable adjunct to his household. Some nights later, when the fireman had his "day off," and was allowed to spend the night at home, in the middle of the night he heard a great commotion in an adjoining room. He leaped from his bed, turned on the light, rushed into the other room and to his utter consternation saw the rattlesnake with his body securely coiled about a burglar, his head wrapped around a bed post and his tail out of the window, rattling for a policeman!

This spirit of generosity has characterized Atlanta through all her years. She has not builded her greatness alone from native sons and daughters, but the Atlanta Spirit, Atlanta's enterprise, Atlanta's courage and Atlanta's accomplishments, won, first the admiration, then the praise and, ultimately, many of the best people in Georgia and neighboring states as permanent citizens. There has been built up a great city from the select men the enterprisng men, the successful men of every vocation and everywhere in Georgia. When restrained by local environment, when impeded by lack of facilities, these aggressive, progressive men have come to Atlanta from far and near, until Atlanta has become indeed and in truth, the Georgia city.

The fame of Atlanta's success has spread both far and wide. Her name is the synonym of progress, her motto is ever onward and upward. Her people are a people who do something.

We have more good things in Atlanta than any other city in the state, more churches, more schools, more mercantile establishments, more banks, more, and a greater diversity, of manufacturing enterprises, more skilled workmen in every phase of industry. We make more doctors and as many lawyers, as the balance of Georgia. Our drummers are the livest wires and the most indefatigable liars on earth. I welcome you as an humble citizen of the city and in the name of all the loyal people who compose it. We rejoice at your coming, we hail with joy your presence, we stretch forth our arms in loving embrace and bid you welcome to our city, to our homes, and to our hearth. Out of the fullness of hearts that are glad we give you the dearest, sweetest welcome we know.

